# The Effect of Vitamin D Supplementation on its Metabolism and the Vitamin D Metabolite Ratio

**DOI:** 10.3390/nu11102539

**Published:** 2019-10-21

**Authors:** Vito Francic, Stan R. Ursem, Niek F. Dirks, Martin H. Keppel, Verena Theiler-Schwetz, Christian Trummer, Marlene Pandis, Valentin Borzan, Martin R. Grübler, Nicolas D. Verheyen, Winfried März, Andreas Tomaschitz, Stefan Pilz, Annemieke C. Heijboer, Barbara Obermayer-Pietsch

**Affiliations:** 1 Division of Endocrinology and Diabetology, Endocrinology Lab Platform, Department of Internal Medicine, Medical University of Graz, 8036 Graz, Austria; vito.francic@medunigraz.at (V.F.); verena.schwetz@medunigraz.at (V.T.-S.); christian.trummer@medunigraz.at (C.T.); marlene.pandis@medunigraz.at (M.P.); valentin.borzan@medunigraz.at (V.B.); martin.gruebler@gmx.net (M.R.G.); stefan.pilz@chello.at (S.P.); 2 Department of Clinical Chemistry, Endocrine Laboratory, Amsterdam Gastroenterology & Metabolism, Vrije Universiteit Amsterdam and University of Amsterdam, Amsterdam UMC, 1105 Amsterdam, The Netherlands; s.ursem@amsterdamumc.nl (S.R.U.); n.dirks@amsterdamumc.nl (N.F.D.); a.heijboer@amsterdamumc.nl (A.C.H.); 3 Department of Laboratory Medicine, Paracelsus Medical University Salzburg, 5020 Salzburg, Austria; keppel.martin@gmail.com; 4 Division of Cardiology, Department of Internal Medicine, Medical University of Graz, 8036 Graz, Austria; nicolas.verheyen@medunigraz.at; 5 Synlab Academy, Synlab Holding Germany GmbH, 68163 Mannheim, Germany; Winfried.Maerz@synlab.com; 6 Health Center Trofaiach-Gössgrabenstrasse, 8739 Trofaiach, Austria; andreas.tomaschitz@gmx.at

**Keywords:** vitamin D metabolites, vitamin D supplementation, vitamin D metabolite ratio, randomized controlled trial, 24,25-dihydroxy vitamin D

## Abstract

25-hydroxyvitamin D (25(OH)D) is commonly measured to assess vitamin D status. Other vitamin D metabolites such as 24,25-dihydroxyvitamin D (24,25(OH)_2_D) provide additional insights into vitamin D status or metabolism. Earlier studies suggested that the vitamin D metabolite ratio (VMR), calculated as 24,25(OH)_2_D/25(OH)D, could predict the 25(OH)D increase after vitamin D supplementation. However, the evidence for this additional value is inconclusive. Therefore, our aim was to assess whether the increase in 25(OH)D after supplementation was predicted by the VMR better than baseline 25(OH)D. Plasma samples of 106 individuals (25(OH)D < 75 nmol/L) with hypertension who completed the Styrian Vitamin D Hypertension Trial (NC.T.02136771) were analyzed. Participants received vitamin D (2800 IU daily) or placebo for 8 weeks. The treatment effect (ANCOVA) for 25(OH)D_3_, 24,25(OH)_2_D_3_ and the VMR was 32 nmol/L, 3.3 nmol/L and 0.015 (all *p* < 0.001), respectively. Baseline 25(OH)D_3_ and 24,25(OH)_2_D_3_ predicted the change in 25(OH)D_3_ with comparable strength and magnitude. Correlation and regression analysis showed that the VMR did not predict the change in 25(OH)D_3_. Therefore, our data do not support routine measurement of 24,25(OH)_2_D_3_ in order to individually optimize the dosage of vitamin D supplementation. Our data also suggest that activity of 24-hydroxylase increases after vitamin D supplementation.

## 1. Introduction

Vitamin D plays an essential role in calcium and phosphate homeostasis [1]. Vitamin D status is most commonly assessed by determining the 25-hydroxyvitamin D (25(OH)D) concentration in serum or plasma. However, several other vitamin D-related metabolites can be measured to provide a better understanding of individual vitamin D status and metabolism. Among them, 24,25(OH)_2_D has emerged as a metabolite with potentially high utility [2].

**Figure 1:**
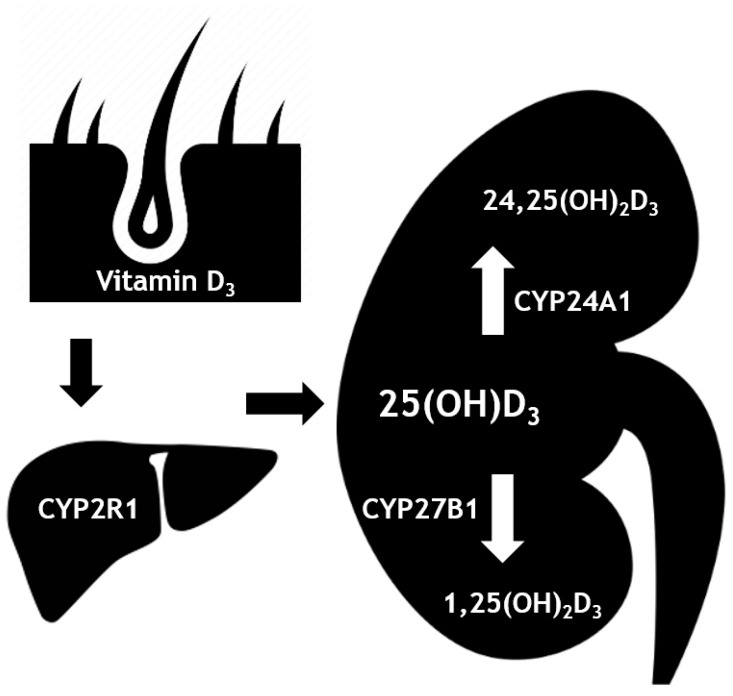
Metabolism of vitamin D. Vitamin D_3_ (cholecalciferol) is produced in the skin when exposed to sunlight. The hepatic enzyme CYP2R1 then converts this into 25(OH)D_3_ (calcifediol). In the kidneys, 25(OH)D_3_ can be converted into the active form, 1,25(OH)_2_D_3_ (calcitriol), by CYP27B1 (1-α-hydroxylase). In the kidneys, CYP24A1 (24-hydroxylase) can catabolize the 25(OH)D_3_ into 24,25(OH)_2_D_3._

In the kidneys, 25(OH)D is converted by 1-alpha-hydroxylase (CYP27B1) into 1,25-dihydroxyvitamin D (1,25(OH)_2_D; also called active vitamin D or calcitriol) (Figure 1). 1,25(OH)_2_D can bind to the vitamin D receptor (VDR) with high affinity. The subsequent signaling results in an increase in serum calcium and phosphate concentrations, mainly mediated by an increased intestinal uptake. In addition, 1,25(OH)_2_D has effects on the parathyroid gland, kidneys and bones, all resulting in an increase in serum calcium and phosphate concentrations [1]. Furthermore, 1,25(OH)2D has major effects on modulating the immune system, which might be relevant for the treatment of autoimmune diseases, infections, cancer and cardiovascular diseases [3]. An excess of both 1,25(OH)_2_D and/or 25(OH)D lead to their catabolism by the enzyme 24-hydroxylase (CYP24A1). This results in the formation of metabolites 1,24,25(OH)_2_D and 24,25(OH)_2_D, respectively [4]. It is still unclear whether 24,25(OH)_2_D has a physiological role in humans [5].

Using an LC-MS/MS method, 25(OH)D and 24,25(OH)_2_D can be measured simultaneously, which allows for determination of the 24,25(OH)_2_D/25(OH)D ratio, also known as the vitamin D metabolite ratio (VMR) [2]. The VMR is an indicator of CYP24A1 activity and thereby of vitamin D catabolism. It is currently used for diagnosing idiopathic infantile hypercalcemia, a rare genetic disorder in which a mutation in CYP24A1 results in severe hypercalcemia and suppressed parathyroid hormone (PTH) levels [5]. The VMR may also reflect vitamin D receptor (VDR) activity since CYP24A1 expression is upregulated in response to 1,25(OH)_2_D [2].

In recent years, there has been an increasing interest in the use of the VMR when assessing vitamin D status. For example, it has been postulated to better reflect vitamin D deficiency [6]. In addition, it has been speculated that the ratio could provide useful information regarding bone health [7]. Interestingly, several studies show that the VMR can predict the change seen in 25(OH)D after vitamin D supplementation, although results are inconclusive [6,8,9,10,11]. The CYP24A1 activity could be partially responsible for the individual differences seen in the effect of vitamin D supplementation on serum levels of 25(OH)D. Theoretically, if CYP24A1 activity is a major predictor of the effect of vitamin D supplementation, the VMR could be used to personalize the treatment dosage. At present, 25(OH)D concentrations at the start of supplementation, as well as BMI, age, ethnicity and genetic background have been most commonly studied in regard to predicting the response to vitamin D supplementation, and studies involving 1,25(OH)_2_D, 24,25(OH)_2_D, free and bioavailable 25(OH)D and the VMR are scarce [12].

Therefore, we set out to determine whether baseline VMR measurements can predict changes in vitamin D-related metabolite levels after vitamin D supplementation. To that extent, we measured 25(OH)D_3_, 1,25(OH)_2_D and 24,25(OH)_2_D_3_ in a randomized clinical trial of patients (25(OH)D < 75 nmol/L) receiving vitamin D supplementation [13]. We hypothesized that measurements of baseline VMR would be advantageous over baseline 25(OH)D measurements for the prediction of the change in 25(OH)D upon supplementation.

## 2. Materials and Methods

### 2.1. Study Cohort

The present post-hoc analysis was conducted in adults (>18 years old) with 25(OH)D levels <75 nmol/L and hypertension, who completed the randomized, placebo-controlled Styrian Vitamin D Hypertension Trial (NC.T.02136771). The participants of this trial were treated with either placebo or 2800 IU daily of vitamin D_3_ (Oleovit D_3_, Fresenius Kabi, Graz, Austria) for 8 weeks. A total of 188 study participants completed the original study and sufficient material for analysis from both study visits was available for 106 of these subjects. The details regarding the study, including inclusion and exclusion criteria, can be found in the publication of the original study by Pilz et al. [13].

Study participants provided written informed consent. The study complied with the Declaration of Helsinki and was approved by the ethics committee of the Medical University of Graz, Austria.

### 2.2. Measurements

For the original study by Pilz et al, the 25(OH)D levels were determined with the ChemiLuminescence assay (IDS-iSYS 25-hydroxyvitamin D assay; Immunodiagnostic Systems Ltd., Boldon, UK) on an IDS-iSYS multidiscipline automated analyser [13]. The intra- and inter-assay CVs were 6.2% and 11.6%, respectively.

In the present study, 25(OH)D_3_ and 24,25(OH)_2_D_3_ were measured in plasma samples by isotope dilution liquid chromatography-tandem mass spectrometry at the Endocrine Laboratory of the Amsterdam UMC, as described previously [14]. For 25(OH)D_3_, the lower limit of quantitation (LLOQ) was 1.2 nmol/L and the inter- and intra-assay coefficients of variation (CV) were 6% and 3%, respectively. For 24,25(OH)_2_D_3_, the LLOQ was 0.1 nmol/L and the inter- and intra-assay coefficients of variation (CV) were 9% and 5%, respectively. 25(OH)D_2_ was also measured, but as the concentrations were all very low (<7.9 nmol/L) and supplementation was given as vitamin D_3_, these data were not taken into account in this paper. In order to calculate the VMR and as it is the golden standard, the LC/MS-MS method was used for the current study. Using this method, 7 subjects had 25(OH)D levels >75 nmol/L at baseline. Measurements of other study parameters have been described previously [13].

To calculate free and biologically available 25(OH)D_3_ we used the equations from Powe et al. [15].

### 2.3. Statistical Analysis

Continuous data following a normal distribution are reported as means with standard deviations (SD). Variables with a skewed distribution are shown as medians with interquartile ranges. Categorical variables are shown as percentages of observations. Groups at baseline were compared using the unpaired Students t-test, the Mann–Whitney U test or the chi-squared test. Skewed variables were log transformed before being used in parametric analyses. 

The changes from baseline for 25(OH)D_3_, 1,25(OH)_2_D and 24,25(OH)_2_D_3_ in the vitamin-D-treated group were calculated as the difference between the value at the final study visit and the value at baseline. They are depicted as Δ25(OH)D_3_, Δ1,25(OH)_2_D and Δ24,25(OH)_2_D_3_. The VMR was calculated as the ratio between 24,25(OH)_2_D_3_ and 25(OH)D_3_. 

Analysis of covariance (ANCOVA) was used to calculate the treatment effects with adjustment for baseline values. Pearson correlation analysis was used to determine the strength of associations between vitamin-D-related parameters and Δ25(OH)D_3_, Δ1,25(OH)_2_D, as well as Δ24,25(OH)_2_D_3_. Bonferroni correction was applied to account for multiple testing. Univariate linear regression analysis was used to determine the relation between Δ25(OH)D_3_ and baseline 25(OH)D_3_, 24,25(OH)_2_D_3_ and VMR.

Using the LC/MS-MS method, 7 subjects had 25(OH)D levels >75 nmol/L at baseline. Therefore, we explored whether inclusion of these subjects had an effect on the analyses. In addition, we also investigated whether the inclusion of only subjects with 25(OH)D levels <50 nmol/L at baseline would affect the analyses.

If outliers were detected in the analyses by the software, defined as cases with standardized residuals greater than 3 standard deviations for ANCOVA analyses or as cases with values higher or lower than 1.5*IQR (interquartile range) for correlation analyses, they were removed and the analysis repeated to determine their potential effect on the analysis. In the case of Pearson correlation analyses, one extreme outlier was removed (25(OH)D > 4xSD at baseline) because of its significant effect on all of the analyses. This is marked in the results section. If the outliers had no significant effect on the analysis, the results including the outliers are reported. A *p*-value < 0.05 was considered statistically significant. All analyses were performed using S.P.SS version 25 (S.P.SS, Chicago, IL, USA).

## 3. Results

The baseline characteristics of study participants can be found in Table 1. There were no differences between the placebo and vitamin-D-treated groups in any of the parameters at baseline.

The calculated treatment effects after vitamin D supplementation are depicted in Table 2. We observed significant treatment effects for all included vitamin-D-related parameters. For 25(OH)D_3_, the treatment effect was 32 nmol/L (95% CI: 26 to 39; *p* < 0.001), for 1,25(OH)_2_D 26 pmol/L (9 to 42; *p* = 0.003), for 24,25(OH)_2_D_3_ 3.3 nmol/L (2.7 to 3.9; *p* < 0.001), for the VMR 0.015 (nmol/L)/(nmol/L) (0.010–0.019; *p* < 0.001), for calculated free 25(OH)D_3_ 12 pmol/L (6 to 18; *p* < 0.001), for calculated bioavailable 25(OH)D_3_ 4.66 nmol/L (2.63 to 6.68; *p* < 0.001), for the 1,25(OH)_2_D/25(OH)D_3_ ratio −0.0010 (nmol/L)/(nmol/L) (−0.0013 to −0.0006; *p* < 0.001) and for the 1,25(OH)_2_D/24,25(OH)_2_D_3_ ratio −0.020 (nmol/L)/(nmol/L) (−0.026 to −0.015; *p* < 0.001). In the subgroup of subjects with 25(OH)D_3_ levels below 50 nmol/L, the treatment effects and *p*-values were comparable for all parameters.

**Figure 2:**
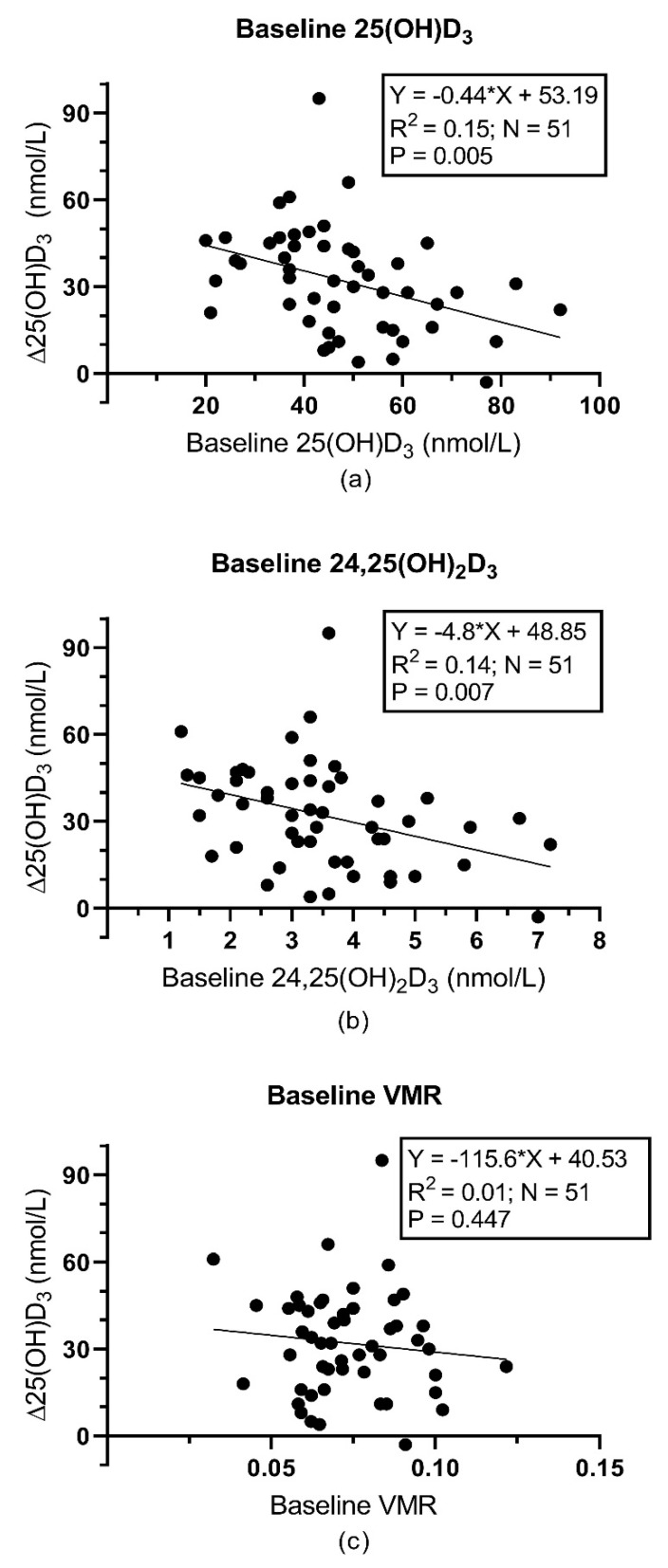
Univariate linear regression analysis for the change in 25(OH)D_3_ concentration in the vitamin D intervention group and (**a**) baseline 25(OH)D_3_, (**b**) baseline 24,25(OH)_2_D_3_ and (**c**) baseline VMR (Vitamin D Metabolite Ratio).

The overall correlation between 25(OH)D_3_ and 24,25(OH)_2_D_3_ at baseline was r = 0.815, *p* < 0.001. Results of the regression analyses of the Δ25(OH)D_3_ in the vitamin-D-supplemented group are shown in Figure 2. The slope of the linear regression, *p*-values and R^2^ values are highly similar for baseline 25(OH)D_3_ and 24,25(OH)_2_D_3_. The VMR, however, could not predict the increase in 25(OH)D_3_ concentration. The results of the correlation analyses in the vitamin-D-treated group are summarized in Table 3. None of the vitamin-D-related parameters correlated significantly with Δ25(OH)D_3_ or Δ1,25(OH)_2_D after Bonferroni correction. Also, in the subgroup of subjects with 25(OH)D levels below 50 nmol/L, none of the parameters correlated significantly with Δ25(OH)D_3_ or Δ1,25(OH)_2_D after Bonferroni correction. For Δ25(OH)D_3_, a trend was seen for baseline 25(OH)D_3_ and baseline 24,25(OH)_2_D_3_ (r = −0.388, *p* = 0.056 and r = −0.374, *p* = 0.056). This trend with Δ25(OH)D_3_ was also observed for calculated free 25(OH)D_3_ and calculated bioavailable 25(OH)D_3_ (r = −0.373, *p* = 0.056 and r = −0.375, *p* = 0.056). Δ24,25(OH)_2_D_3_ was significantly associated with baseline 25(OH)D_3_, 24,25(OH)_2_D_3_, calculated free 25(OH)D_3_ and calculated bioavailable 25(OH)D_3_ (r = −0.562, *p* < 0.001; r = −0.476, *p* = 0.003; r = −0.382, *p* = 0.048 and r = −0.393, *p* = 0.032, respectively), but not with other parameters. In the subgroup of subjects with 25(OH)D_3_ levels below 50 nmol/L, none of the parameters correlated significantly with Δ24,25(OH)_2_D_3_ after Bonferroni correction.

Correlation analyses after adjustment for gender, age, BMI, PTH, eGFR, serum phosphate and serum calcium showed that none of the vitamin-D-related parameters were significantly associated with Δ25(OH)D_3_ or Δ1,25(OH)_2_D after Bonferroni correction (Table A1). However, when corrected for the above-mentioned parameters, only baseline 25(OH)D_3_ was still significantly associated with Δ24,25(OH)_2_D_3_ (r = −0.657, *p* = 0.008). In the subgroup of subjects with 25(OH)D_3_ levels below 50 nmol/L, none of the parameters correlated significantly with Δ25(OH)D_3_, Δ1,25(OH)_2_D or Δ24,25(OH)_2_D_3_ after Bonferroni correction.

## 4. Discussion

The goal of our study was to assess whether vitamin D metabolites can predict the increase of 25(OH)D after vitamin D supplementation. As elaborated above, CYP24A1 activity (24-hydroxylase) is reflected by the ratio of 24,25(OH)_2_D over 25(OH)D, i.e. the VMR. In addition, the ratio between 1,25(OH)_2_D and 24,25(OH)_2_D_3_ was recently proposed as part of a three-dimensional model for assessing vitamin D metabolic pathways [16]. It was previously suggested that vitamin D metabolites and their ratios could provide additional information for predicting vitamin D treatment response [8,9]. The findings in this vitamin D RC.T. in patients with 25(OH)D levels <75 nmol/L and hypertension do not support this hypothesis. 

In our study, the VMR did not predict Δ25(OH)D_3_ in the treatment arm of the RC.T.. In a regression model, baseline 24,25(OH)_2_D_3_ and baseline 25(OH)D_3_ did, with comparable strength and magnitude, predict the increase in 25(OH)D_3_ upon treatment. When adjusting for multiple testing in correlation analyses, no correlations of any of the included parameters with Δ25(OH)D_3_ retained significance. Yet, we did observe trends for Δ25(OH)D_3_ with baseline 25(OH)D_3_, 24,25(OH)_2_D_3_, free 25(OH)D_3_ and bioavailable 25(OH)D_3_. Notwithstanding their borderline significance, the strength of the correlations is highly similar between these parameters and they do not seem to be superior to baseline 25(OH)D. According to these data, we can infer that CYP24A1 activity, measured by the VMR, does not predict the individual differences in the increase in 25(OH)D after vitamin D supplementation.

Concerning the VMR, the results of this study are in accordance with several other published reports. Saleh et al. performed an RC.T. of 4 weeks with 107 participants receiving a single 100,000 IU dose of vitamin D or placebo [11]. The VMR could not predict the increase of 25(OH)D after 4 weeks, whereas 25(OH)D did predict this increase with a similar R^2^-value to our data. However, their data indicated that 24,25(OH)_2_D_3_ could not predict the Δ25(OH)D_3_, whereas in our study it did. Aloia et al. reported on the predictive properties of the VMR in four different small samples (between 14 and 16 participants per group) of placebo or 800, 2000 or 4000 IU vitamin D daily for 10 weeks [6]. They did not show an advantage of the VMR as a predictor, compared to baseline 25(OH)D, 24,25(OH)_2_D_3_ or free 25(OH)D. Binkley et al. investigated the effect of 1800IU of vitamin D in 62 postmenopausal women after 4 months and measured vitamin D metabolites [10]. They observed that neither VMR, 25(OH)D, 24,25(OH)_2_D_3_ nor free 25(OH)D was related to the observed increase in 25(OH)D.

On the contrary, other published studies did suggest a predictive role for the VMR. The study by Wagner et al. included young adults with a mean age of around 27 years that received 28,000 IU (equivalent to 4000 IU per day) of vitamin D once per week for 8 weeks in the form of a supplement or fortified cheese. Wagner et al. showed that the VMR predicted the increase in vitamin-D-receiving subjects (R^2^ = −0.38, *p* = 0.004, *n* = 60) [8]. Also, Cashman et al. reported a significant correlation between the VMR and the change after vitamin D supplementation (R^2^ = 0.15, *p* < 0.01) in a study including subjects above 50 years of age that were treated for 15 weeks by 20 µg vitamin D (800 IU) per day [9]. Of note, both studies did not report the R^2^-value of baseline 25(OH)D with its increase after supplementation. Therefore, it is not possible to conclude whether the VMR was superior to 25(OH)D in this aspect.

Changes in other vitamin-D-related parameters after vitamin D treatment were also studied. To that end, we assessed if Δ1,25(OH)_2_D and Δ24,25(OH)_2_D_3_ could be predicted by baseline parameters included in the study. We found no correlation between any tested baseline parameter and Δ1,25(OH)_2_D. 1,25(OH)_2_D levels are mainly regulated by calcium levels, which could explain this observation [12]. On the other hand, baseline 25(OH)D_3_, 24,25(OH)_2_D_3_, calculated free 25(OH)D_3_ and calculated bioavailable 25(OH)D_3_ all showed a significant correlation with Δ24,25(OH)_2_D_3_. The clinical relevance of this observation is, in our opinion, unclear and should be further studied.

In our study, we observed an increase in the VMR upon vitamin D treatment. This suggests an increase in CYP24A1 activity and catabolism of 25(OH)D upon supplementation. A concurrent decrease in the 1,25(OH)_2_D/25(OH)D_3_ ratio implies a reduced conversion of 25(OH)D to 1,25(OH)_2_D. Indeed, this suggests the physiological shift from anabolic to catabolic pathways when an excess of vitamin D exists. This is also supported by the significant decrease in the 1,25(OH)_2_D/24,25(OH)_2_D_3_ ratio. In the present study, and all aforementioned studies, the correlation coefficients between baseline 25(OH)D and Δ25(OH)D_3_ after supplementation were negative, which implies that the change in 25(OH)D_3_ after vitamin D treatment is smaller in individuals with higher baseline 25(OH)D_3_ levels [8,9,10].

We acknowledge that this study has several limitations. First, the results are derived from post-hoc analyses. Second, the study population consisted of hypertensive subjects with 25(OH)D levels <75 nmol/L; therefore, the findings might not be readily extrapolated to the general population. Furthermore, for the vitamin D level inclusion criterion, the 25(OH)D concentrations were measured at study baseline using a chemiluminescence assay, while mass-spectrometry-based methods are currently the gold standard [2]. However, for the current study, 25(OH)D and 24,25(OH)_2_D were re-measured using a dedicated LC-MS/MS method. In addition, the intervention period of 8 weeks was relatively short and only a small number of subjects were severely vitamin D deficient. Vitamin D deficiency was defined as a 25(OH)D of <75 nmol/L in the original study by Pilz et al. [13]. There is still an ongoing debate as to whether the cut-off levels should be set at <50 nmol/L or <75 nmol/L [17,18]. In addition, vitamin D sufficiency was defined by measurements of baseline 25(OH)D_3_, which is currently the critical measurement for defining vitamin D status [19]. Some studies suggest that free 25(OH)D_3_ could be a better marker for assessing vitamin D status [20]. In our study, calculated free 25(OH)D_3_ did not predict Δ25(OH)D3 after supplementation better than baseline 25(OH)D_3_. The RC.T. design and the successful vitamin D intervention are strengths of this study. Also, a high number of parameters were measured with gold-standard methods. In contrast to the majority of exploratory studies on the VMR, *p*-values of the correlations were adjusted for multiple testing.

In summary, we show that 25(OH)D_3_, 24,25(OH)_2_D_3_ and the VMR increase after vitamin D treatment. However, 24,25(OH)_2_D_3_ and the VMR could not predict 25(OH)D_3_ levels after vitamin D treatment in this cohort better than baseline 25(OH)D_3_. As this has been corroborated by other studies, it implicates the routine measurement of 24,25(OH)_2_D_3_ will probably be of no added value when personalizing the treatment dosage of vitamin D.

## Figures and Tables

**Table 1:** Baseline characteristics.

Parameter	All (*n* = 106)	Placebo (*n* = 54)	Vitamin D (*n* = 52)	*p*-value
Age (years)	62.0 (51.3–68.7)	64.8 (50.8–70.2)	59.6 (52.4–66.6)	0.318
Body mass index (kg/m^2^)	30.0 ± 5.4	29.7 ± 5.9	30.3 ± 4.9	0.562
Gender (% female)	57	57	56	0.865
24,25(OH)_2_D_3_ (nmol/L)	3.5 ± 1.6	3.4 ± 1.5	3.6 ± 1.5	0.419
25(OH)D_3_ (nmol/L)	48 ± 18	46 ± 19	49 ± 18	0.401
VMR ((nmol/L)/(nmol/L))	0.073 ± 0.017	0.072 ± 0.018	0.073 ± 0.017	0.768
PTH (pmol/L)	5.5 (4.1–6.7)	5.5 (4.0–6.7)	5.3 (4.1–6.7)	0.779
1,25(OH)_2_D (pmol/L)	126 ± 53	118 ± 52	133 ± 52	0.142
Serum phosphate (mmol/L)	0.94 ± 0.17	0.96 ± 0.17	0.92 ± 0.16	0.282
Serum calcium (mmol/L)	2.26 (2.21–2.33)	2.26 (2.21–2.34)	2.26 (2.20–2.33)	0.773
eGFR (mL/min/1.73m^2^)	72 ± 17	69 ± 16	74 ± 18	0.152
24h urinary calcium excretion (mmol/24h)	3.30 (1.90–5.00)	2.95 (1.83–4.78)	3.70 (2.10–6.30)	0.222
Calculated free 25(OH)D_3_ (pmol/L)	15 (9–21)	12 (8–21)	17 (11–20)	0.153
Vitamin D binding protein (µg/mL)	247.1 ± 109.5	254.8 ± 110.6	239.3 ± 109.0	0.772
Calculated bioavailable 25(OH)D_3_ (nmol/L)	5.9 (3.9–8.2)	5.2 (3.2–8.5)	6.6 (4.1–8.0)	0.149
1,25(OH)_2_D /25(OH)D_3_ ((nmol/L)/(nmol/L))	0.0023 (0.0019–0.0036)	0.0027 (0.0018–0.0039)	0.0028 (0.0021–0.0035)	0.753
1,25(OH)_2_D /24,25(OH)_2_D_3_ ((nmol/L)/(nmol/L))	0.036 (0.025–0.05)	0.036 (0.024–0.051)	0.035 (0.026–0.050)	0.893

**Table 2:** Analysis of covariance (ANCOVA) analysis for the effect of vitamin D or placebo treatment on vitamin-D-related parameters.

Parameter	Group	Baseline	Follow-up	Treatment Effect (95% CI)	*p*-value
25(OH)D_3_ (nmol/L)	Placebo, *n*=54	46 ± 19	45 ± 20	32 (26 to 39)	<0.001
	Vitamin D, *n*=52	49 ± 18	79 ± 19		
1,25(OH)_2_D (pmol/L)	Placebo, *n*=52	118 ± 52	114 ± 39	26 (9 to 42)	0.003
	Vitamin D, *n*=52	133 ± 52	150 ± 63		
24,25(OH)_2_D_3_ (nmol/L)	Placebo, *n*=54	3.4 ± 1.5	3.3 ± 1.8	3.3 (2.7 to 3.9)	<0.001
	Vitamin D, *n*=52	3.6 ± 1.6	6.8 ± 1.7		
VMR	Placebo, *n*=54	0.072 ± 0.018	0.071 ± 0.017	0.015 (0.010 to 0.020)	<0.001
	Vitamin D, *n*=52	0.073 ± 0.017	0.087 ± 0.018		
Calculated free 25(OH)D_3_ (pmol/L)*	Placebo, *n*=53	12 (8–21)	12 (8–18)	12 (6 to 18)	<0.001
	Vitamin D, *n*=51	17 (11–20)	21 (17–31)		
Calculated bioavailable 25(OH)D_3_ (nmol/L) *	Placebo, *n*=53	5.22 (3.15–8.51)	4.99 (2.95–6.83)	4.66 (2.63 to 6.68)	<0.001
	Vitamin D, *n*=51	6.60 (4.10–8.02)	8.69 (6.58–12.51)		
1,25(OH)_2_D/ 25(OH)D_3_ *	Placebo, *n*=52	0.0027 (0.0018–0.0039)	0.0026 (0.0019–0.0036)	−0.0010 (−0.0013 to −0.0006)	<0.001
	Vitamin D, *n*=52	0.0028 (0.0021–0.0035)	0.0019 (0.0014–0.0026)		
1,25(OH)_2_D /24,25(OH)_2_D_3_ *	Placebo, *n*=52	0.036 (0.024–0.051)	0.037 (0.026–0.052)	−0.020 (−0.026 to −-0.015)	<0.001
	Vitamin D, *n*=52	0.035 (0.026–0.050)	0.022 (0.016–0.028)		

* Log-transformed parameters.

**Table 3:** Pearson correlations with unadjusted *p*-values and Bonferroni adjusted *p*-values of baseline vitamin-D-related parameters with the changes from baseline of 25(OH)D_3_, 1,25(OH)_2_D and 24,25(OH)_2_D_3_ after vitamin D supplementation.

Baseline Parameters		Δ25(OH)D_3_	Δ1,25(OH)_2_D	Δ24,25(OH)_2_D_3_
25(OH)D_3_	r	−0.388	−0.142	−0.562
	*p*-value	0.005	0.322	<0.001
	Adjusted *p*-value	0.056	1.000	<0.001
1,25(OH)_2_D	r	−0.287	−0.260	−0.272
	*p*-value	0.041	0.065	0.053
	Adjusted *p*-value	0.328	0.520	0.424
24,25(OH)_2_D_3_	r	−0.374	−0.122	−0.476
	*p*-value	0.007	0.392	<0.001
	Adjusted *p*-value	0.056	1.000	0.003
VMR	r	−0.109	−0.027	−0.015
	*p*-value	0.448	0.850	0.916
	Adjusted *p*-value	1.000	1.000	1.000
Calculated free 25(OH)D_3_ *	r	−0.373	−0.281	−0.382
	*p*-value	0.007	0.046	0.006
	Adjusted *p*-value	0.056	0.368	0.048
Calculated bioavailable 25(OH)D_3_ *	r	−0.375	−0.280	−0.393
	*p*-value	0.007	0.047	0.004
	Adjusted *p*-value	0.056	0.376	0.032
1,25(OH)_2_D/25(OH)D_3_ *	r	−0.004	−0.058	0.176
	*p*-value	0.980	0.687	0.216
	Adjusted *p*-value	1.000	1.000	1.000
1,25(OH)_2_D /24,25(OH)_2_D_3_ *	r	0.053	−0.028	0.181
	*p*-value	0.711	0.843	0.204
	Adjusted *p*-value	1.000	1.000	1.000

* Log-transformed parameters.

## Appendix A

**Table A1:** Pearson correlations of baseline vitamin-D-related parameters adjusted for gender, age, BMI, PTH, eGFR, serum phosphate and serum calcium, with the changes from baseline of 25(OH)D, 1,25(OH)_2_D and 24,25(OH)_2_D after vitamin D supplementation. *p*-values without and with Bonferroni adjustment are shown.

Baseline Parameters		Δ25(OH)D_3_	Δ1,25(OH)_2_D	Δ24,25(OH)_2_D_3_
25(OH)D_3_	r	−0.508	−0.277	−0.657
	*p*-value	0.013	0.201	0.001
	Adjusted *p*-value	0.104	1.000	0.008
1,25(OH)_2_D	r	−0.350	−0.171	−0.430
	*p*-value	0.102	0.435	0.040
	Adjusted *p*-value	0.816	1.000	0.320
24,25(OH)_2_D_3_	r	−0.490	−0.129	−0.597
	*p*-value	0.018	0.559	0.003
	Adjusted *p*-value	0.440	1.000	0.096
VMR	r	−0.064	0.137	−0.516
	*p*-value	0.773	0.534	0.012
	Adjusted *p*-value	1.000	1.000	0.096
Calculated free 25(OH)D_3_ *	r	−0.451	−0.363	−0.399
	*p*-value	0.031	0.089	0.059
	Adjusted *p*-value	0.248	0.712	0.472
Calculated bioavailable 25(OH)D_3_ *	r	−0.451	−0.363	−0.404
	*p*-value	0.031	0.089	0.056
	Adjusted *p*-value	0.248	0.712	0.448
1,25(OH)_2_D/25(OH)D_3_ *	r	0.122	0.272	0.218
	*p*-value	0.578	0.209	0.318
	Adjusted *p*-value	1.000	1.000	1.000
1,25(OH)_2_D /24,25(OH)_2_D_3_ *	r	0.126	0.136	0.211
	*p*-value	0.565	0.536	0.333
	Adjusted *p*-value	1.000	1.000	1.000

* Log-transformed parameters.
